# Magnetic Springs

**Published:** 1870-07

**Authors:** 


### *Magnetic Springs.*

Prof. Robert C. Kedzie, M.D., of Lansing, Mich., is inclined to attribute the magnetic peculiarities of the much vaunted St. Louis and other mineral springs, of that State, to the iron tubing of the artesian wells through which the water flows. Various speculations are afloat on the subject, and meanwhile the springs are haunted by multitudes of the lame, halt, and blind, and cures are as abundant as whilom in this city from the commands of "Prof. Newton." His "Disease, depart!" only equaled in efficacy the waters with "Magnetism blown into the bottles" of the Michigan pools of Bethesda. *Populus vult decipi, et decipiatur.*

